# Editorial: DNA & RNA methylation: impact on cancer progression

**DOI:** 10.3389/fgene.2023.1293897

**Published:** 2023-09-27

**Authors:** Vaibhav Shukla, Ashish Tyagi

**Affiliations:** Irma Lerma Rangel School of Pharmacy, Texas A&M University, College Station, TX, United States

**Keywords:** DNA methylation, RNA methylation, non-coding RNAs, cancer, RNA modification

The complexity of genomes does not only involve the composition of billions of base pairs but also includes chemical modification of bases through epigenetic mechanisms. Among all epigenetic modifications, DNA methylation has been most widely studied since 1948 when it was first reported (Hotchkiss, 1948). In humans, DNA methylation is regulated by a family of enzymes, DNA methyltransferases (DNMTs), which covalently add methyl groups to cytosine residues predominantly at CpG sites (Bourchis et al., 2001). It plays an important role in normal homeostasis including regulation of transcription (van Eijk et al., 2012), embryonic development (Smith et al., 2014), chromatin structure, and genome stability (Vilain et al., 2000). With the advancement in technology, aberrant DNA methylation has been observed in many disease conditions including cancer (Baier, 1988). Regulation by non-coding RNA has been also shown to function as a modulator of epigenetics. Among all non-coding RNA, microRNAs (miRNAs) affect the protein levels of target genes without changing the gene sequences and are the subject of intensive ongoing investigations (Peng and Croce, 2016). Similar to conventional DNA methylation, diverse chemical modifications of cellular RNAs have also emerged as regulators of gene expression. This includes N6-methyladenosine (m6A), 7-methylguanosine (m7G), and more. RNA modification has been shown to play a key role in tumorigenesis and the progression of various cancers (Song et al., 2022). This Research Topic aimed to summarize the underlying molecular mechanism of DNA and RNA modification in human diseases including cancer. This will pave the way for the development of new strategies or methods for disease prevention, diagnosis, and therapy. In total, 2 original research articles and 1 review article has been published in this Research Topic.

Gutierrez-Angulo et al. provide a review on the Research Topic focusing on the role of microbiota in DNA methylation in colorectal cancer. They have shown the association of gram-negative and positive bacteria with DNA hypermethylation of tumor suppressor genes such as MLH1, and MTSS1. They have reported that high levels of pathogenic bacteria *Fusobacterium* nucleatum have been associated with the development, prognosis, and treatment response for colorectal cancer patients. Authors in this article believed that changes in gut microbiota have the potential to modulate gene expression via DNA methylation and aid in colorectal cancer prognosis.

The second article by Wang et al. demonstrated the link between RNA methylation and the formation of tumor microenvironment (TME) in hepatocellular carcinoma. They have performed m7G modification evaluation in 819 hepatocellular carcinoma specimens from the TCGA-LIHC cohort, the LIRI-JP cohort in ICGC, and the GSE14520 cohort from the GEO database. They identified different m7G modification patterns and correlated them with the infiltration characteristics of immunocytes. They also found the NUDT16 gene was significantly altered and had high CNV among tumor tissues. The authors concluded that the m7G methylation pattern played an important role in the formation of TME and may improve immunotherapy efficacy.

An article by Liu et al. has shed light on altered circular RNAs (circRNAs) involved in colorectal cancer (CRC) pathogenesis and metastasis. They performed differential expression of circRNAs using GEO datasets for colorectal cancer specimens and validated the expression of selected circRNAs using qRT-PCR. They found the upregulation of hsa-circ-0040809 and hsa-circ-0000467 in CRC tissues and cell lines. Further, they performed *in vitro* functional analysis such as CRC cell migration, and proliferation for the two upregulated circRNAs. They also constructed a circRNA-miRNA-mRNA network. The authors emphasized the potential role of these axes in CRC tumorigenesis and prognosis.

In summary, by taking advantage of different public datasets, published reports, and validation experiments, authors have reported the role of epigenetics including DNA methylation, RNA methylation, and circRNA-miRNA-mRNA axes in different cancers. We would like to express our gratitude to all the authors contributing to this Research Topic. These studies advance our knowledge on the function of DNA and RNA methylation and non-coding RNAs in human diseases and paved the way for future work in the field. However, more samples and validation techniques will be needed in the future to strengthen the findings shown in the articles. We hope our study Research Topic will help us to learn more about DNA and RNA methylation.
